# A Pruning Method for Deep Convolutional Network Based on Heat Map Generation Metrics

**DOI:** 10.3390/s22052022

**Published:** 2022-03-04

**Authors:** Wenli Zhang, Ning Wang, Kaizhen Chen, Yuxin Liu, Tingsong Zhao

**Affiliations:** Faculty of Information Technology, Beijing University of Technology, Beijing 100124, China; wangn@emails.bjut.edu.cn (N.W.); kzchen@emails.bjut.edu.cn (K.C.); liuyuxin1998@emails.bjut.edu.cn (Y.L.); zhaotingsong@emails.bjut.edu.cn (T.Z.)

**Keywords:** pruning network, redundant features, layers

## Abstract

With the development of deep learning, researchers design deep network structures in order to extract rich high-level semantic information. Nowadays, most popular algorithms are designed based on the complexity of visible image features. However, compared with visible image features, infrared image features are more homogeneous, and the application of deep networks is prone to extracting redundant features. Therefore, it is important to prune the network layers where redundant features are extracted. Therefore, this paper proposes a pruning method for deep convolutional network based on heat map generation metrics. The ‘network layer performance evaluation metrics’ are obtained from the number of pixel activations in the heat map. The network layer with the lowest ‘network layer performance evaluation metrics’ is pruned. To address the problem that the simultaneous deletion of multiple structures may result in incorrect pruning, the Alternating training and self-pruning strategy is proposed. Using a cyclic process of pruning each model once and retraining the pruned model to reduce the incorrect pruning of network layers. The experimental results show that proposed method in this paper improved the performance of CSPDarknet, Darknet and Resnet.

## 1. Introduction

With the widespread use of computer vision, deep learning networks based on multimodal data (visible images (RGB images), depth images, infrared images) are applied to various fields, including object detection [1,2,3,4,5] and classification [6,7], image segmentation [8,9,10], target tracking [11,12,13], etc. While most of the mainstream algorithms are designed based on RGB images and show better performance when extracting key features from RGB images. However, the amount of effective information contained in an infrared image is not balanced with that of an RGB image, which has a single feature and does not contain fine-grained information such as and texture. Related studies have shown [14,15] that the networks used to extract RGB image features are not suitable for direct use to extract key features in infrared images. Generally, the deeper the network layers of a deep learning model, the better the image feature extraction. However, for images such as infrared images, which only have simple features such as edge contours, applying a deeper network to extract features will produce the problem of over-fitting. The network layers are prone to extracting redundant information. Therefore, the network layers where redundant features are extracted need to be pruned to improve the network’s ability to extract key features from images and reduce the loss of important features. To achieve this, the researcher uses the following network pruning approach to the mainstream algorithm. Today there are two main types of research into network pruning methods: one is aimed at lightweight the network and improving the speed of network operations. The other is used to enhance the network’s ability to capture key features in images. The first type of pruning method assesses the strengths and weaknesses of the network structure through internal network parameters and prunes the network structure based on the assessment results. Some researchers [16,17,18,19,20,21] have applied the importance of the gradient propagation or loss value of the network to measure the structure of the network. Ref. [22] determine whether the pruning is correct by comparing the output of the network structure with the output of the pruned network. Refs. [23,24] used a scaling factor of the Batch Normalization function to calculate the contribution of the network structure to the final results. The second type of pruning method focuses on the subjective assessment of good or bad network structures through visual images [25,26,27]. Pruning the network structures that contribute less.

The above network pruning methods have the following problems.

**Figure 1:**
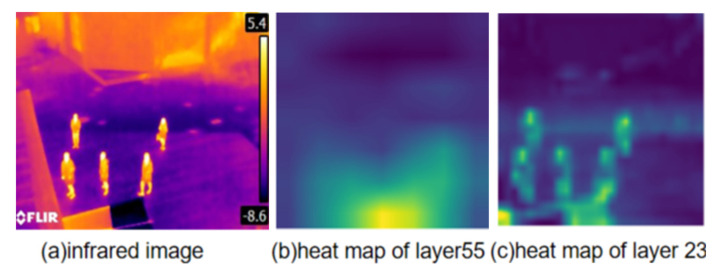
Figure (**a**) shows the infrared image used for visualization, Figure (**b**) shows the heat map obtained from network layer 55 and Figure (**c**) shows the heat map visualized from network layer 23, the area beyond the pedestrian in the Figure is the background area.

1. The pruning method for the purpose of network lightweight applies the scaling factor parameter of the normalization function in the deep learning model, KL scatter and other evaluation metrics to quantify the contribution of the network layers to the final result, and cannot evaluate the network feature extraction performance. It cannot improve the feature extraction ability of the network.
2. Deep learning networks are known to be uninterpretable in terms of feature extraction at each layer. Although visualization tools can visualize the extraction of target and background features in an image by means of a heat map, it is not possible to describe what kind of features are extracted at each layer of the network and whether they are effective. However, it is not possible to describe from the visualization what features are extracted at each layer of the network and whether the extracted features are valid. This makes it impossible to determine how well the network layer is able to extract key features and whether it should be cut down. Figure 1b,c show a visualization of the features extracted by the yolov4 target detection network at different layers.
3. The existing network pruning methods use a pre-trained model to train the network and fine-tune the pruned network model. However, this causes the initial parameters of the network to change, so that the parameters of each network layer are not at the same standard when the network is trained. When performing the network layer evaluation, each network layer is influenced by the parameters of the pre-trained model. The values of the corresponding network layer evaluation metrics also change.

This paper investigates the above issues based on a pruning approach to improve network performance. Using infrared images as the research object, to enhance the feature extraction capability of the network layer and to quantify the feature extraction performance of the network layer, a pruning method for deep convolutional network models based on heat map generation metrics is proposed. In order to extract the key features of infrared images. The main contributions of this paper are as follows.

1. This paper addresses the problem that some researchers are prone to misjudge the performance of network layers by observing visual images with the human eye or judging the feature extraction ability of the network layers by experience. In this paper, we propose a formula that can quantitatively describe the feature extraction performance of each network layer, including ‘foreground feature extraction capability metrics’ (*F*), ‘background feature suppression capability metrics’ (*B*), ‘network layer performance evaluation metrics’ (*NPE*). By objectively calculating the feature extraction capability of a network layer. Pruning of network layers to reduce incorrect pruning due to subjective errors in judgement.
2. To address the problem that traditional network pruning methods fine-tune pre-trained models, which are prone to incorrect pruning. In this paper, we propose an alternating training and self-pruning strategy. Only the worst performing network layers in the model are pruned at a time, and the network is trained from zero. The operation of alternating network pruning and training from zero is repeated to improve the quality of extracted features.

The experiments show that the proposed method of pruning deep convolutional models based on heat map generation metrics can quantitatively evaluate the performance of network layers. The method achieves the function of accurate redundant network layer pruning. The alternate training and self-pruning strategy is used to prune the network, which saves time and achieves the effect of automatic pruning. The paper presents related work in Section 2, the pruning method is described in detail in Section 3, the relevant experiments performed and results are discussed in Section 4, the conclusions and shortcomings of the proposed method and future improvements are presented in Section 5.

## 2. Related Work

With the rapid development and widespread use of deep learning, two problems have gradually emerged: one is that the number of network layers is too deep and the amount of computation is too large. It leads to the inability to deploy the network on edge devices. The other is the different complexity of image features for different modalities. The same network extracts feature from different modalities, which tends to produce inadequate feature extraction or feature loss. To address these two problems, researchers have made the following studies.

In response to the first category of questions, Liu et al. [16] proposed a discrimination-aware channel pruning algorithm. The method inputs a trained network model and introduces additional discrimination-aware losses to increase the discrimination capability of the intermediate layer. Considers the discrimination-aware loss error and reconstruction error to select the most discriminative channel for each layer. Each iteration prunes the unimportant channels of the network layer to achieve the purpose of network pruning. Yu et al. [17] proposed a method for evaluating networks using neuron importance propagation scores. Applying an infinite feature selection method (Inf-FS) to evaluate the importance of the last layer of responses. Calculating the importance scores of all neurons by back propagation, pruning the channels with lower importance until the pruning rate is reached; Zeng W et al. [21] proposed a unified and automatic pruning framework which focuses on applying the Hessian approximation to assess the importance of components. It can be used to identify the unimportant components of the network, which can be used for refinement and structured pruning; Luo et al. [22] proposed a method for pruning filters. Takes the output of the layer i and a subset of the layer i output are used as the input to the layer i+1. If the two layer i+1 outputs are approximately equal, then the filters corresponding to channels other than the subset in the layer i output are all pruned. Liu et al. [23] proposed a method to calculate the pruning rate applying the Batch Normalization layer. Since it is a common operation to add a Batch Normalization layer after the convolution. Each Batch Normalization layer contains γ parameter, this paper applies the γ parameter as the filter evaluation score. Removes the filter when the γ parameter is close to zero. Luo et al. [24] proposed a way to evaluate the pruned network using KL -divergence criterion. By using the γ parameter in the Batch Normalization layer as the coefficient of each channel in the network layer. Removing the channels with γ close to 0. The output of the network before and after pruned is saved. the similarity of the two network outputs is evaluated using the KL scatter criterion; the more similar the two network outputs are, the more accurately the redundant channels are pruned.

In response to the second category of questions, Zhang et al. [25,26] used a visualization tool to transform the output of each network layer into a heat map image. The feature extraction capability of the network layer was then judged by visual observation of the extracted contours of the foreground targets in the output heat map of each network layer. If the target contour features are found to be incomplete, the network layer is judged to be weak in feature extraction and is thus pruned. Zhang et al. [27] proposed an improved yolov3 for vehicle detection in infrared images. To better extract key features in infrared images, the network layers with poor feature extraction performance were pruned with experience, and the 53-layer network in the original yolov3 was pruned to 16 layers. The quality of feature extraction was improved.

## 3. Methods

### 3.1. Overview

To address the problem of unsatisfactory pruning due to subjective observations. We propose computational formulas and network pruning strategies that can quantitatively describe the feature extraction performance of each layer of the network. There are two issues to be considered in the network pruning process:

Problem 1: The performance of the pruned network will not be improved and may even be degraded. Whether the performance of the network can be improved depends on two aspects: First, whether the key features are extracted during the training process, i.e., the target features in the image. Second, whether the background is well suppressed during the training process, i.e., the features are suppressed in non-target region. Therefore, this paper proposes the *NPE*. *NPE* is calculated by using *F* and *B*. This paper uses the *NPE* to determine how well the network layer extracts the features of the test image. 

Problem 2: Most traditional methods prune the pre-trained model and fine-tune the pre-trained model. The same network layer performs differently before and after model pruning. If the pruned model is fine-tuned on top of the pre-trained model, the pruned network will be influenced by the parameters of the pre-trained model when evaluating the network layer. This can lead to inaccurate results in the evaluation of network layer performance. Therefore, this paper proposes a strategy of alternating between pruning and training networks, i.e., alternate training and self-pruning strategy.

**Figure 2:**
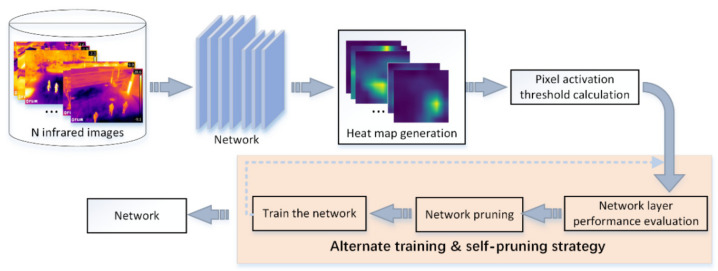
Network pruning method.

This chapter focuses on the method of network structure pruning. Aiming to prune a network that is suitable for extracting infrared image features. The network layers are visualized by a visualization tool, on the basis of which the network layer performance is assessed. The visualization tools include CAM [28], Grad-CAM [29], Grad-CAM++ [30], Score-CAM [31] etc. CAM, Grad-CAM, Grad-CAM++, can only capture a single target. However, in practical applications where there are more targets in a 2D image, only the Score-CAM visualization method is able to capture multiple targets in the image. Therefore, the visualization tool in this paper applies Score-CAM to generate a heat map for each layer of the network. In addition, the heat map is normalized. The specific network pruning process is shown in Figure 2. Firstly, an infrared image is input into the network model. The heat map is obtained by applying Score-CAM. By setting different pixel activation thresholds, the output heat map of the network layer in the middle position is used to calculate its *F* and *B*. When *F* and *B* are approximately equal, the pixel value is selected as the pixel activation threshold. Then the pixel activation threshold is used to calculate the network layer metrics, including *F*, *B* and *NPE*. *NPE* is then used to evaluate the performance of the network layer. Cycle through N infrared images in order to calculate the ‘mean network layer performance evaluation metrics’ (*mNPE*). Secondly, the worst performing network layers are pruned. Finally, the pruned network is trained from zero, cycling through the operations of pruning the network and training the network from zero.

### 3.2. Heat Map Based NPE Construction Method

By observing the heat map of each network layer, it is found that the feature extraction of each network layer is different. The brighter the position indicates its higher activation level and the better the feature extraction. As shown in Figure 1, showing the heat map of network layer 55 and network layer 23 in yolov4 [6]. By observing Figure 1, few pedestrians are activated in Figure 1b, and relatively more pixel points are activated in the background. In Figure 1c, the pedestrians are basically activated in the heat map, and the number of activated pixel points in the background is relatively small. However, the activated pixel points cannot be clearly identified by human eye observation only, and the performance of the network layer cannot be accurately assessed. Therefore, this paper applies a pixel activation threshold to clearly define the activated and inactivated pixels. In this paper, we calculate the *F* and *B* by the number of pixel activation. So as to use the *F* and the *B* to calculate the *NPE*. The following section describes the acquisition of the pixel activation threshold, compute *F*, compute *B* and compute *NPE*.

#### 3.2.1. Compute F

**Figure 3:**
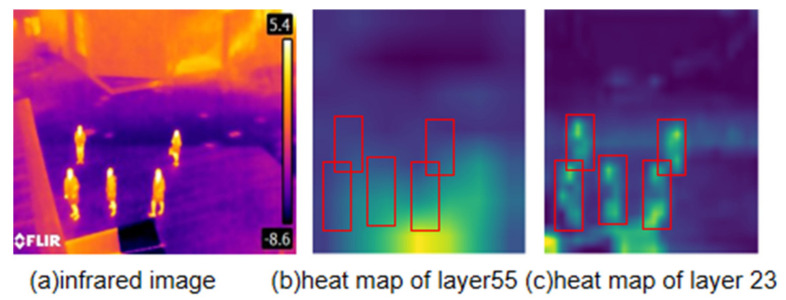
Heat map analysis. Figure (**a**) shows the infrared image used for visualization, Figure (**b**) shows the heat map obtained from network layer 55, and Figure (**c**) shows the heat map visualized from network layer 23, with the area beyond the pedestrian in the figure as the background area. The red boxes are manually labelled true value boxes.

The network can extract and learn object features to better distinguish between different objects, which is the basis of object detection or classification. Therefore, a good extraction of object features at the network layer is crucial to the task of target detection or classification. As shown in Figure 3. This paper uses the mean value of the pixels in the ground truth to compare with the pixel activation threshold to determine whether the pedestrian is activated or not. Judging the object extraction ability of the network by the proportion of activated pedestrians to all pedestrians. Based on this, this paper proposes *F* to quantify the feature extraction capability of the network. The calculation steps are as follows:

Firstly, the annotated ground truth is mapped to the feature map output of the network layer. The average value of pixel points in the ground truth is calculated, that is the average pixel point value of each person (*Average_Person*). As shown in (1), where represents the sum of all pixel point values in the ground truth, and *n_point* represents the number of pixel points in the truth frame. Secondly, the pixel point activation threshold is compared with *Average_Person* to determine whether the person is activated. The number of activated pedestrians in the image is counted (*Exact_Person*). Finally, *F* is calculated, as shown in (2), where *All_Person* indicates the number of all pedestrians in the image.

(1) $$Average_{Person}=\frac{\sum_{Groundtruth}point}{n\_point}$$

(2) $$F=\frac{Exact\_Person}{All\_Person}$$

#### 3.2.2. Compute B

Background redundant information can affect the network’s ability to learn target features. This causes the network to learn incorrect information and reduces the accuracy of the target detection or classification task. It is therefore important to evaluate the suppression of background redundant information, as shown in Figure 3. The area outside the ground truth in the image is the background area. The calculation steps are as follows:

Firstly, the background information after pruning the ground truth is input. The total number of pixels in the background is calculated. Secondly, all the pixels in the background are compared with the pixel activation threshold. The pixels that are smaller than the pixel activation threshold is counted cumulatively, that is the pixels that are inactivated. The ratio of the number of inactive pixels in the background (*back_point*) to the total number of pixels in the background (*back_allpoint*) is used to calculate the *B*, as shown in (3).

(3) $$B=\frac{Back\_point}{Back\_allpoint}$$

#### 3.2.3. Compute NPE

The feature extraction ability of the network layer depends mainly on the extraction of target features and the suppression of background features. The extraction of background features interferes with the target features. Both the background feature suppression capability and the target feature extraction capability have an impact on the comprehensive performance of the network model. Therefore, *NPE* is calculated using *F* and the *B*. 

The larger *F* and the higher *B*, represents the better object feature extraction capability of the network layer and the higher the *NPE*. However, with the selection of the pixel activation threshold, *F* and *B* showed a negative correlation; In the evaluation of the object detection algorithm, the larger precision, the higher recall, the better the detection performance of the network is indicated. However, with different values of the class score threshold, the evaluation indicators Precision and Recall also show a negative correlation trend. In order to evaluate the detection performance of the object detection algorithm, the detection precision and the recall were applied to calculate the comprehensive evaluation metrics F1_measure. The comprehensive evaluation metrics F1_measure was used to evaluate the detection performance of the network. The trends of the *F* and *B* are consistent with the trends of the detection precision and the detection recall. Therefore, *NPE* is designed with F1_measure. Its calculation formula is shown in (4), thus defining *F*, *B* and *NPE*.

(4) $$NPE=\frac{2*F*B}{F+B}$$

When a single image is input to the pruning method proposed in this paper, a set of evaluation metrics can be obtained for each network layer, namely *F*, *B* and *NPE*. Since there may be no pedestrian target in the randomly selected image. In addition, *F* is 0, that is *NPE* is also 0, which will incorrectly assume that the network layer performance is poor. Therefore, in order to reduce the error caused by applying a single image to evaluate a network layer, this paper applies n images to evaluate each network layer. Calculates n sets of evaluation metrics, and applies the average of n *NPE* as the performance score of the network layer, that is ‘average network layer performance evaluation metrics’ (*mNPE*). n can be selected for different values depending on the situation. However, the larger the n value is, the larger the n value is, the greater the number of input infrared images. It is more convincing to evaluate the performance of each layer of the network in different background infrared images. However, at the same time, the longer it takes to prune each layer of the network. Conversely the smaller the n value, the smaller the number of input images. Evaluating network layers with an average performance evaluation metric is prone to chance. This can lead to incorrect evaluation results and incorrect pruning of network layers, For the purposes of this paper, n is taken to be the empirical value of 16.

#### 3.2.4. Obtain the Pixel Activation Threshold

How to effectively distinguish foreground target features extracted by convolutional neural networks from background features remains a problem. We believe that in a deep network model, if the foreground target feature extraction ability of that network layer is better and the background feature suppression ability is stronger, then the feature extraction ability of that network layer is higher. However, in reality, the target feature extraction ability and the background feature suppression ability are negatively correlated, so there is a balance point between the target feature extraction ability and the background feature suppression ability. How to find the equilibrium point is a key issue. In this paper, we propose a method for selecting the activation threshold of pixel points.

**Figure 4:**
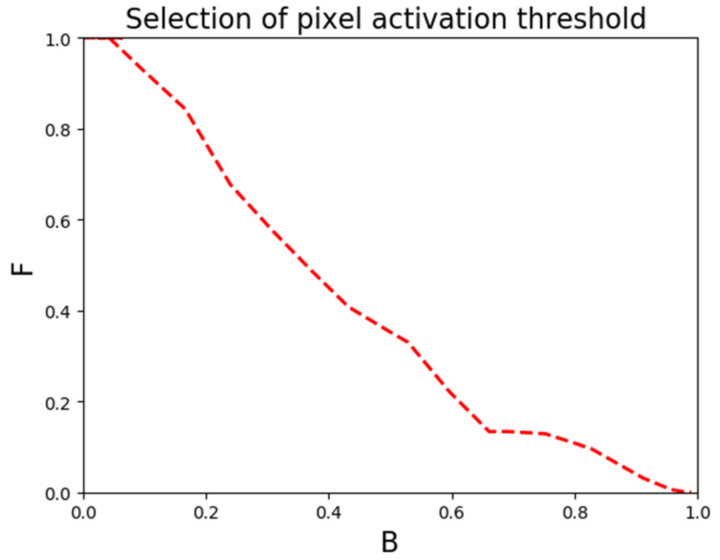
Selection of pixel activation threshold.

The heat map is normalized to a value of 0–1 for each pixel. With higher values indicating better feature extraction and brighter pixels. When the threshold is set too high, the number of activated targets is reduced. In addition, *F* is underestimated, *B* is overestimated. When the threshold is set too low, the number of activated pixels in the image is incorrectly assumed to increase. In addition, *F* is overestimated, *B* is underestimated. It is important to set the appropriate pixel activation threshold. As shown in Equation (4), when *F* and *B* are approximately equal, *NPE* is the highest, that is the performance of the network layer is evaluated to be the best. Therefore, in this paper, we experimentally set different pixel activation thresholds M in the range of 0–1. As shown in Figure 4. When the pixel activation threshold is set to 0.2, *F* is approximately equal to *B*. Therefore, the pixel activation threshold is selected as 0.2.

### 3.3. Alternating Training and Self-Pruning Strategy

**Figure 5:**
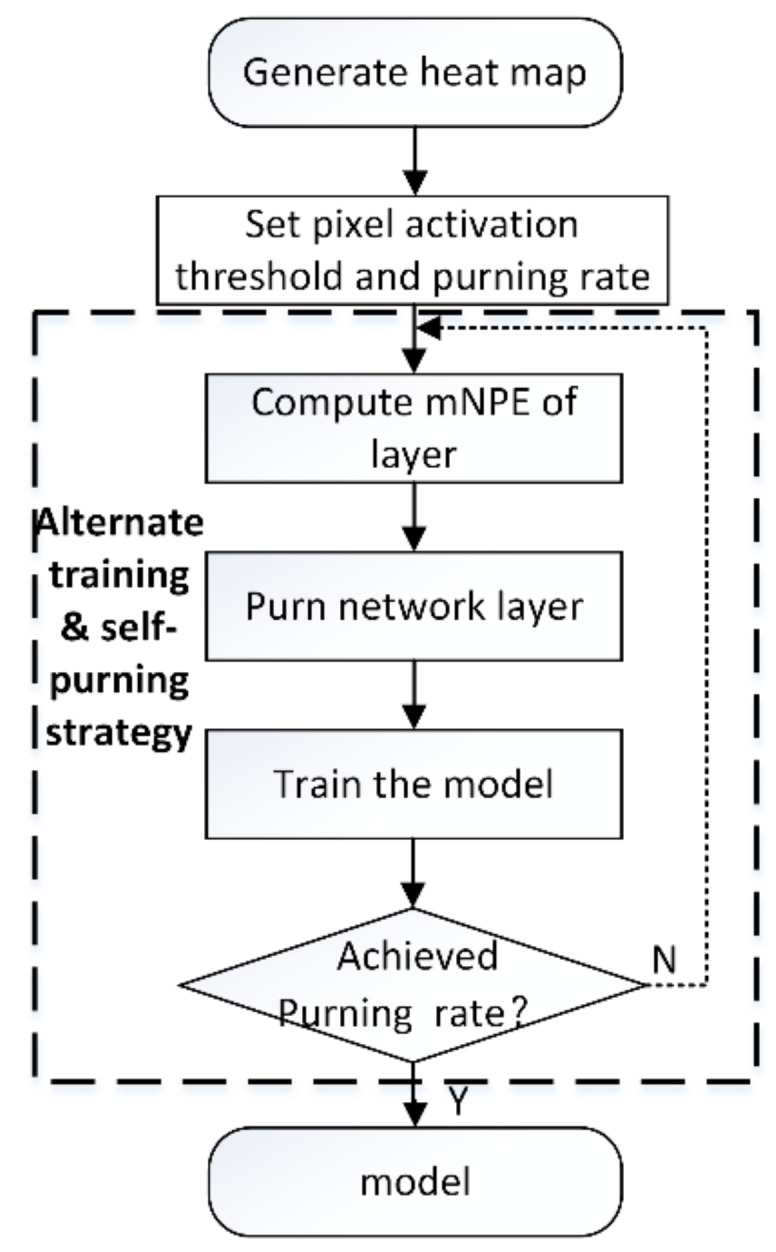
Alternating training and self-pruning strategies.

The completion of tasks such as object detection, object tracking and object classification is attributed to the construction of the network. Since forward and backward propagation is performed during network training, when a network layer in the network is changed, the parameters of each network will also change during the propagation process. The performance of other network layers is affected. In addition, if the model is not retrained at this time, the performance of each network layer after the change cannot be viewed in a timely manner. Therefore, in order to view the changes in the performance of network layers in a timely manner, this paper proposes an alternate training and self-pruning strategy for pruning the network, as shown in Figure 5.

(1) Heat map generation. The Score-CAM algorithm is applied to obtain a heat map of the network layer that you want to evaluate.
(2) Set the pixel activation threshold and pruning rate. Pixel activation thresholds are selected when *F* and *B* are approximately equal. The pruning rate is the ratio of the number of layers pruned to the total number of layers in the model. The number of layers to prune is determined by the pruning rate. In this paper, 10%, 20% and 30% are chosen as the pruning rates.
(3) Calculate the *mNPE* of the network layer. When there is no target in the input image, the performance evaluation index of the network layer is all 0. It is impossible to compare the performance of the network layer. In order to reduce the chance of the input image, input *n* infrared images. Each network layer in the model to obtain *n* *NPE*, and calculate the average of *n* *NPE* as *mNPE* of the network layer.

Prune and train the network. The network layer with the lowest *mNPE* is pruned and the pruned network is retrained. Finally, alternate between pruning the network layers and training the model until the pruning rate is reached.

## 4. Results

### 4.1. Overview

This chapter begins with details of the experimental environment and dataset, including the dataset, experimental environment and experimental evaluation metrics. Secondly, as the object detection task is widely used, it is found that the network is too deep will have a bad impact on the extraction of infrared image features and thus reduce the detection accuracy. This paper uses three popular deep convolutional models, including CSPDarknet, Darknet and ResNet. Pruning the network using the proposed pruning method. The proposed pruning method is applied to the object detection task to verify the effectiveness of this pruning method.

### 4.2. Experimental Details

#### 4.2.1. Datasets

Infrared images have simple features such as edge contours. Ordinary deep learning models will have over-fitting problems due to the deeper layers of the network. In addition, our team is conducting research related to object detection based on infrared images. Therefore, this paper uses infrared images as the research object. For different modal images, such as depth images, which also have simple features such as edge contours, the network pruning method in this paper can also be applied. However, it is not the object of study in this paper and this extension will be carried out in future studies. In the infrared image dataset applied in this paper, GTOT is the public dataset and school is the dataset we took ourselves. We use the public dataset to verify the generality of the method. Our own photographed dataset demonstrates the practicality of the method.

GTOT: This dataset is derived from Li et al. [32]. It took in daytime and nighttime scenes. Specific scenes include office areas, public roads and pools, etc., with a total of 50 video sequences. This paper mainly uses video sequences from public road scenes to detect pedestrians walking near the road, with a total of 1329 infrared images. The dataset has the characteristics of small scale and density of pedestrians. The proportion of pedestrians in the images is small. It is difficult to extract the key features.

School: This dataset is a self-took dataset. It is mainly erected at a height of 5–6 m, with the camera lens at 30–45° to the ground. It was taken in the daytime and nighttime scenes. The scene mainly including the school canteen, the school building, the flyover and outside the gym door, etc. This dataset has a total of 1943 infrared images. It records three types of pedestrian movement: queuing, grouping and discussion. The queuing scene is mainly included pedestrian queuing move horizontally and pedestrian queuing move longitudinally. The lateral movement of pedestrians capturing the side faces and the longitudinal movement of pedestrians capturing the front faces. The group scene is mainly included a group move horizontally and a group move longitudinally. This scene mainly simulates an occlusion situation. The discussion scene divides the pedestrians into 2–3 groups. Each group includes 2–3 people. This scene mainly simulates a crowded situation and increases the difficulty of detection.

The two datasets have a total of 3272 infrared images. In this paper, the infrared images are divided into 6:4, of which 1963 images are used for training and 1309 images are used for testing.

#### 4.2.2. Experimental Environment

The proposed method for pruning deep convolutional network based on heat map generation metrics was built by the pytorch framework and the experiment was carried out on a computer equipped with a 3090ti.

#### 4.2.3. Experimental Performance Evaluation Metrics

In this paper, the pruned network is used to the object detection task. The network is judged to be superior or inferior by the result of object detection. In the object detection task, this paper uses map and *F*1-measure to evaluate the pedestrian detection performance of the pruned network which used to judge the pruning accuracy. The map indicates the average detection precision of each class. *F*1-measure can be calculated by Precision and Recall, which trade off the precision and recall. We consider an object to be successfully detected when the overlap between the predicted box and ground truth is greater than 0.6. The metrics are calculated as follows:

(5) $$Precision=\frac{TP}{TP+FP}$$

(6) $$Recall=\frac{TP}{TP+FN}$$

(7) $$F1\_measure=\frac{2*Precision*Recall}{Precision+Recall}$$

where *TP* indicates the number of correctly detected pedestrians, *FP* is the number of false detections and *FN* the number of missed detections.

### 4.3. Discussion

#### 4.3.1. Ablation Experiments

The aim of this experiment is to investigate the feasibility of the proposed network pruning method. The deep network is pruned based on infrared images. The pruned network extracts key features of the targets in the infrared images and applies them to different tasks, such as target detection, target classification and target tracking. The performance of the pruned network is judged by observing the accuracy with which each task is completed. Regardless of the task performed, the labelled images should be used as input when applying the network pruning method in this paper. Yolov4, yolov3, retinanet, are the object detection models that are widely used and have good performance evaluation at this stage. The backbone networks are CSPDarknet, Darknet and Resnet152, which have deeper layers, 104, 74 and 152, respectively. Deeper networks can improve the object detection capability. However, when the number of network layers is too deep, the problem of over-fitting will easily arise. Therefore, CSPDarknet, Darknet and ResNet are chosen as the object of network pruning in this paper. As CSPDarknet, Darknet, Resnet152, etc. are composed of residual blocks, the residual blocks are composed of convolutions. Therefore, in this paper, the residual blocks in the network are pruned. The performance of each residual block is evaluated by the last layer’s mNPE of the network output.

**Figure 6:**
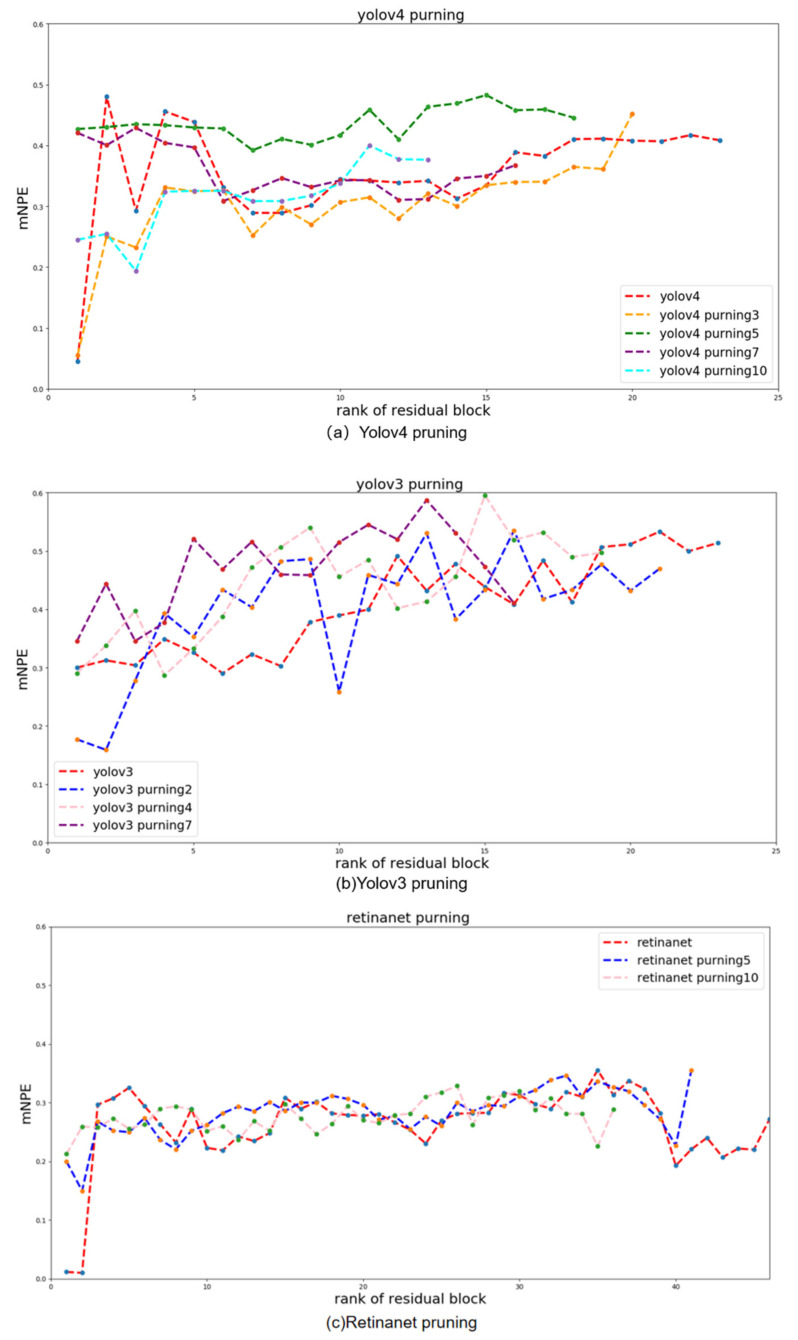
The pruning process, with the horizontal coordinates of the residual block number and the vertical coordinates of the overall evaluation index of the residual block. Figure (**a**) shows the pruning process of yolov4, Figure (**b**) shows the pruning process of retinanet, Figure (**c**) shows the pruning process of yolov3.

Network pruning process: The horizontal coordinate in Figure 6 is the residual block number and the vertical coordinate is the comprehensive evaluation index of the residual block. This is used to evaluate the performance of this residual block. A higher *mNPE* value indicates a better residual block performance, i.e., more critical information can be extracted. Each line represents a pruning model. Yolov4 in Figure 6a indicates the evaluation of 23 residual blocks of yolov4, and each node indicates the evaluation result of the corresponding residual block. The first residual block was found to be the worst performing in the yolov4 residual block evaluation results. Yolov4 pruning1 indicates the worst performing residual block in the pruned model of yolov4. Since the first residual block in the yolov4 residual block evaluation curve has the worst performance. Therefore, the first residual block in the yolov4 model needs to be pruned. Yolov4 pruning1 is the first residual block to be pruned on top of yolov4. Yolov4 pruningN is the worst residual block to be pruned in the yolov4 pruningN-1 model. Yolov3 and retinanet networks are pruned in the same way.

**Figure 7:**
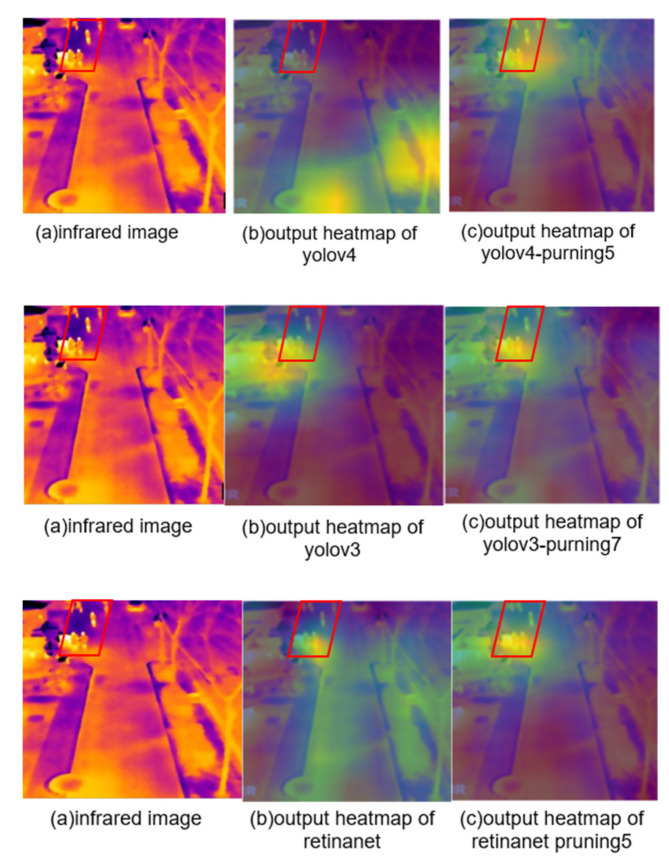
Presentation of feature visualization results. Figure (**a**) is the original input image. Figure (**b**) shows the results of the network feature visualization before pruning. Figure (**c**) shows the results of the network feature visualization after pruning.

We found that the map value of the object detection model generally decreased when the pruning rate reached 30% through several groups of experiments. Therefore, we believe that a pruning rate higher than 30% is not useful for improving the feature extraction ability of the network. Therefore, we chose to adopt an equal interval approach to select the pruning rate with a 30% threshold, and adjusted the pruning rate according to the different performance of the network. Yolov4 pruning rates were set to 10%, 15%, 20% and 30% in turn. Yolov3 pruning rates were set to 10%, 20% and 30% in turn. Retinanet pruning rates were set to 10% and 20% in turn. In Figure 6a–c shows the pruning process of yolov4, yolov3 and retinanet networks, respectively. From the Figure 6, it can be seen that when the yolov4, yolov3 and retinanet pruning rates are 15%, 30% and 10%, respectively, i.e., yolov4_pruning5, yolov3_pruning7 and retinanet_pruning5, the mNPE values of the network are all higher. The performance of the network is best at this time. Figure 7a–c shows the final feature map visualization results for each network before and after pruning, respectively. Based on the visualization results, it can be observed that the pruned networks extract more pedestrian features than the pre-pruning networks. Therefore, the proposed network pruning metrics and pruning strategy are more accurate in pruning the network structure that extracts redundant features. The performance of the networks in performing each task is also improved.

Pruned network performance verification: To validate the findings above, all models were tested for object detection on the GTOT dataset and the school dataset collected by myself. As shown in Table 1. 13 models were validated in this paper, including yolov4, yolov3, retinanet and their pruned networks. The pruning rates are shown in the table.

As shown in Table 1 and Figure 5, when the mNPE of the last layer of the network in each residual block of the network model is high, the more key features are extracted. The better the performance achieved in performing the task. The more layers of the network model are pruned, the faster the pruned network performs the task.

#### 4.3.2. Comparison Experiments

The model in Table 2 indicates the lightweight classification network model. GFLOPs indicates the amount of computation when the network is run after pruning. Error indicates the error rate when the pruned network performs classification. These parameters are chosen in this paper to accurately represent the change in computation during the pruning process and the impact of the pruning on the classification task. The comparison methods listed [16,17,18,19,24,25] are the better network pruning methods for recent research results. Since the Taylor_pruning pruning method has been compared with [16,17,19,20,24,25], Taylor_Pruning has the best performance and produces the least error. Therefore, the proposed pruning method in this paper was compared with Taylor_Pruning.

The proposed method is mainly used to improve the network’s ability to capture key features in images. The merits of the pruning method are judged by the object detection performance of the network. The comparison results found that when the same pruning rate was set, as shown in Table 3, the target detection results obtained by applying the two different pruning methods were different. The proposed network pruning method in this paper yields higher map values for the object detection algorithm. It shows that among the 10% of the network channels pruned by the Taylor_pruning method, some of the channels were incorrectly pruned without extracting the redundant features. Moreover, the difference between the map values obtained by the Taylor_pruning method and the proposed method is larger as the network deepens. The proposed algorithm is more accurate in pruning the network structure where redundant features are extracted. The method improves the extraction ability of key features of the network, which leads to a good improvement of the object detection performance.

The method proposed in this paper solves the problem that the network model is too deep, resulting in the model extracting to redundant features. The proposed method can achieve accurate pruning of the network structure of the extracted redundant features and obtain a pruned network model suitable for extracting key features of infrared images. At the same time, the proposed method can also be applied to other deep network models to improve the performance of the network model.

In addition, this network pruning method can be applied to a variety of fields, such as object detection, object tracking, object recognition and object classification. Feature extraction is necessary in all of these areas. By applying the method proposed in this paper, the key features extraction capability of the network can be enhanced and the accuracy of different networks performing different tasks can be improved.

## 5. Conclusions

This paper proposes a pruning method for deep convolutional network based on heat map generation metrics, which can be applied to the pruning of network structures in different domain’s network to achieve the extraction of key features in different datasets. Network layer’s performance evaluation metrics, including F, B and mNPE, are proposed for evaluating the network layer performance and pruning the network layers with poor performance. An alternate training and self-pruning strategy is proposed. Reducing the occurrence of incorrectly judged network layer performance due to interference from pre-trained model parameters. The experimental results show that the network pruning method not only improves the network operation speed, but also the performance of the object detection task as the network layers that extract redundant features are pruned to extract key features in the infrared images and retain the high-level semantic information and shallow detail information in the infrared images. In this paper, the mean value of pixel points in ground truth is compared with the pixel point activation threshold to determine whether pedestrians are extracted or not. However, ground truth generally contains more pixel points than pedestrians, and therefore contains some redundant pixel points. In the next step, a Gaussian distribution will be applied to label the pedestrian area. The mean value of the pixel points in the pedestrian area is then calculated instead of calculating the mean value of the pixel points inside the ground truth. In the future, the application of this method will be extended to validate the application to depth images and improve the generality of the pruning method.

## Figures and Tables

**Table 1:** Results of object detection experiments.

Experiment	Network	Precision (%)	Recall (%)	Map (%)	F1 (%)	Time (ms)
Experiment 1	yolov4(Baseline)	0.735	0.885	0.899	0.803	5.7
Experiment 2	yolov4 pruning3	0.738	0.891	0.906	0.807	5.6
Experiment 3	yolov4 pruning5	0.926	0.825	0.914	0.872	5.6
Experiment 4	yolov4 pruning7	0.76	0.893	0.908	0.821	5.5
Experiment 5	yolov4 pruning10	0.907	0.819	0.899	0.861	5.4
Experiment 6	yolov3(Baseline)	0.81	0.918	0.927	0.861	5.5
Experiment 7	yolov3 pruning2	0.951	0.913	0.93	0.93	5.4
Experiment 8	yolov3 pruning4	0.798	0.916	0.925	0.853	4.9
Experiment 9	yolov3 pruning7	0.83	0.92	0.932	0.873	4.6
Experiment 10	retinanet(Baseline)	0.85	0.808	0.807	0.828	78.2
Experiment 11	retinanet pruned5	0.818	0.812	0.861	0.815	70.7
Experiment 12	retinanet pruned10	0.801	0.812	0.837	0.807	64.8

**Table 2:** Classification results evaluate existing cutting methods.

Method	Model	GFLOPs	Params (10^7^)	Error, %
Taylor-FO-BN-72% [18]	ResNet-50	1.34	0.79	28.31
NISP -50-B [17]	ResNet-50	2.29	1.43	27.93
ThiNet-72 [22]	ResNet-50	2.58	1.69	27.96
Taylor-FO-BN-82% [18]	ResNet-34	2.83	1.72	27.17
Li et al. [33]	ResNet-34	2.76	1.93	27.8
Taylor-FO-BN-50% [18]	VGG11-BN	6.93	3.18	30
Slimming [19], from [34]	VGG11-BN	6.93	3.18	31.38

**Table 3:** The detection results were evaluated by the method in this paper and the comparison method.

Method	Model	Precision	Recall	Map	F1	Pruning Rate
Taylor-FO-BN [18]	retinanet	0.86	0.703	0.824	0.774	10%
proposed	retinanet	0.818	0.812	0.861	0.815	10%
Taylor-FO-BN [18]	yolov4	0.76	0.893	0.901	0.821	10%
proposed	yolov4	0.915	0.814	0.908	0.862	10%
Taylor-FO-BN [18]	yolov3	0.917	0.875	0.93	0.896	10%
proposed	yolov3	0.951	0.913	0.93	0.93	10%

## Data Availability

Not applicable.
